# The Evolving Field of Stereotactic Body Radiation Therapy in Pancreatic Cancer

**DOI:** 10.17140/POJ-3-110

**Published:** 2019-11-06

**Authors:** Maged Ghaly, Emile Gogineni, Muhammad W. Saif

**Affiliations:** 1 Department of Radiation Medicine, Northwell Health Cancer Institute, Lake Success, NY, USA; 2 Department of Medical Oncology, Northwell Health Cancer Institute, Lake Success, NY, USA

**Keywords:** Pancreatic neoplasms, Pancreas cancer, Radiosurgery, Stereotactic, Stereotactic body radiation therapy (SBRT), Radiation, Radiotherapy

## Abstract

Pancreatic cancer remains a devastating disease with dismal outcomes despite the development of novel chemotherapeutic regimens and radiation techniques. Stereotactic body radiation therapy (SBRT) offers an advantage both in image guidance and radiation dose delivery to direct ablative doses to tumors with acceptable toxicity compared to conventional techniques. Recent literature is clustered with data pertaining to SBRT in patients with resectable, borderline resectable and locally advanced pancreatic tumors. We here present a summary of the current data and highlight the limitations and potential for future growth. Further clinical study in the form of multi-institutional trials is warranted to establish the role of SBRT in combination with new chemo- therapeutic agents as well as a non-invasive alternative to surgery.

## INTRODUCTION

Pancreatic ductal adenocarcinoma (PDAC) is an aggressive malignancy with limited effective therapeutic options and exceedingly high mortality. Currently, a cure may be achieved through resection; recent evidence suggests that neoadjuvant therapy can increase R0 (pathologically negative margin) resection rates with effective local control.^1^ Stereotactic body radiation therapy (SBRT) has garnered significant interest for pancreatic cancer patients as it is completed quickly over 1–5 fractions, requires less time away from full doses of chemotherapy, and is generally much better tolerated than conventional radiographic testing (RT) as a result of more limited target volumes. Favorable results of SBRT for locally advanced pancreatic cancer (LAPC) patients are now leading to the exploration of SBRT for other pancreatic cancer patients.^2^

### SBRT FOR LOCALLY ADVANCED PANCREAS

The utility of pancreatic SBRT was established in the locally advanced patient population. With the advent of gemcitabine-based (GEM) chemotherapy, the role of RT for LAPC has become more precarious.^3^ The European Fédération Francophone de Cancérologie Digestive (FFCD)/The Société Francophone de Radiothérapie Oncologique (SFRO) Phase III trial compared GEM alone *versus* induction 5 Fluorouracil (FU) and cisplatin chemoradiation (CRT), followed by maintenance gem.^4^ Overall survival (OS) was shortened in the CRT arm from 13 to 8.6-months. Higher grade 3 toxicities with CRT were observed during both induction (36% *vs.* 22%) and maintenance (32% *vs.* 18%) phases. Notably, the trial utilized a higher than normal conventionally fractionated 60 Gy dose. The recent success of more aggressive, but increasingly toxic, chemotherapy regimens such as FOLFIRINOX and gem plus nab-paclitaxel have spurred re-examination of local therapy.^5,6^ With improved systemic control, local progression may become a more serious issue for survival and quality of life. However, local control rates from standard external beam radiotherapy (EBRT) have been disappointing with 1-year local progression rates of around 50%.^7^ Furthermore, with two-thirds of patients failing distantly within 1 year, a shorter course approach with minimal interruption to systemic therapy is desirable.^7^ These factors paved the way for the use of SBRT in pancreatic cancer patients, and initially those with LAPC.

The inception of SBRT for pancreatic cancers began at Stanford with a phase I dose escalation study in a LAPC cohort.^8^ The trial was stopped at a dose of 25 Gy since all patients achieved local control with distant metastasis as the first site of failure. The median survival for all patients was 11-months, with 100% local control. However, despite smaller margins and less acute toxicity, patients treated on the Stanford single-fraction SBRT protocol experienced a high degree of late toxicities (25% grade ≥2).^9^ Hypofractionated studies showed reduced 1-year grade 2 toxicity to 7.8%. This reduction came without a compromise in disease control. The 1-year local control was 91.5% *vs.* 88.3% (*p*=0.8) for single *vs.* 5-fraction SBRT with median OS of 13.6-months for all patients. More contemporary SBRT series have also largely employed a fractionated approach.^10–14^ These institutional studies re- veal a median survival of 14–15-months, 1-year local control rates of about 80%, and grade 3 toxicities below 10%.^15^

Very recently, a few groups have reported that LAPC patients may have an increased likelihood of undergoing resection after aggressive induction chemotherapy regimens. Recently, the group from Hopkins reported on 88 patients treated from 2010–14 with SBRT using gem-based or FOLFIRINOX regimens.^16^ SBRT doses ranged from 25–33 Gy in 5 fractions. The 1-year local control rate was 61%, but with a median OS of 18.4-months for LAPC patients. Notably, 20% of LAPC patients underwent surgery. Resected patients had a median OS of 20.2-months, compared to 12.3-months for unresected cases. Grade 3 toxicity was below 6%. Similar to the study from Hopkins, SBRT data from Moffitt also shows the possibility of downstaging for surgery.^14^ They reported a 24% surgical conversion rate for LAPC patients receiving FOLFIRINOX chemotherapy. All converted patients achieved an R0 (microscopic negative margin) resection. Any grade 3 or higher toxicity was 7%. Median OS was 34.2-months for patients who underwent resection, and 11.3-months for those who did not. See Table 1 for a list of SBRT studies for LAPC.

### SBRT FOR BORDERLINE RESECTABLE PANCREAS

While pancreatic SBRT has been most extensively evaluated in LAPC patients, there is emerging data that SBRT may also benefit patients with borderline resectable pancreas (BRPC) (Table 2). The SBRT literature for BRPC largely comes from the Moffitt Cancer Center. Chuong et al reported on a larger series of 73 patients (57 BRPC, 16 LAPC) who received induction gem, docetaxel, and capecitabine (GTX) followed by SBRT.^12^ SBRT was delivered using 5 consecutive daily fractions targeting the primary tumor with a median dose of 30 Gy (range, 25–30 Gy), the region of vasculature involvement was prescribed a median dose of 35 Gy (range, 35–50 Gy) using a simultaneous integrated boost (SIB) to further increase the likelihood of tumor regression and R0 resection. After restaging, 56.1% of the BRPC patients underwent surgical resection with all except for one (96.9%) having negative margins. Resected patients had significantly improved median OS (19.3 *vs.* 12.3 months; *p*=0.03) and median progression-free survival (PFS) (12.7 *vs.* 5-months; *p*<0.0001). No acute grade 3 toxicities were reported and the most common acute toxicities were grade 1–2 fatigue and nausea. Their subsequent study of 159 patients (110 BRPC, 49 LAPC), surgical resection was performed on 51% of the BRPC patients and R0 resection was achieved in 96%. Portal vein (PV) or superior mesenteric vein (SMV) resection and reconstruction was performed in 34% of BRPC patients. Median OS was significantly higher among patients who had surgery compared to those who did not (34.2 *vs.* 14.0-months; *p*<0.001). Finally, while the prescription doses generally increased compared to the previous publication (primary tumor: median 30 *vs.* 35 Gy; tumor-vessel interface: median 35 *vs.* 40 Gy), the incidence of late grade 3 radiation-related toxicity remained consistently low (~5%).^14^

The feasibility of using SBRT for BRPC is also supported by other studies with more limited numbers of BRPC patients. A study from Johns Hopkins included 88 patients (74 LAPC, 14 BRPC) who received 5-fraction SBRT and reported favorable surgical and SBRT-related toxicity outcomes.^17^

Investigators from the University of Pittsburgh published their experience of 12 patients (7 BRPC, 5 LAPC) who received chemotherapy followed by SBRT prescribed to 36 Gy in 3 fractions (n=7) or 24 Gy in a single fraction (n=5) and then had surgery.^18^ A high rate of R0 resection was achieved (92%) with minimal toxicity. Pathologic complete response (pCR) was achieved in 25%, which is higher than would be expected with standard EBRT and perhaps signaling that SBRT may have unique histopathologic effects. It is plausible that a higher rate of pCR may be achieved using dose fractionation schedules with a higher biologically effective dose. He et al compared surgical outcomes among BRPC/ LAPC patients who received SBRT (n=29), CRT (n=82), or chemotherapy alone (n=26) and reported R0 resection rates of 90%, 84% and 62%, respectively (*p*=0.02).^19^ The PCR rate was notably higher among patients who received SBRT (21% *vs.* 4% *vs.* 0%; *p*<0.001).

In conclusion, while various neoadjuvant treatment regimens are commonly used for BRPC including standard fractionation CRT, increasing consideration should be given to SBRT based on its clear advantage in increasing R0 resectability with higher PCR rates, and providing improved OS in these patients.

### SBRT FOR RESECTABLE PANCREAS

The significance of microscopic margin involvement on survival is a controversial topic, with some studies claiming an impact on survival and others finding no such correlation.^20^ Recent studies based on rigorous pathological examination protocols report R1 rates of well over 70%.^21–25^ Several studies have shown that residual cancer cells are frequently present in the resection bed even in appropriately staged patients after surgery that is properly performed,^26^ where even with R0 resections nearly 80% of patients were found to have evidence of microscopic cells left *in situ* at the surgical site.^27^ In a recent phase III adjuvant chemotherapy trial in patients with resected pancreatic cancer in which many patients had positive margins (0–60%) and nodal involvement (63–80%), local recurrence rates were 18–41%, suggesting the presence of residual disease may benefit from local therapy in addition to systemic therapy.^28^ Early data from MD Anderson Cancer Center included 86 patients who received gemcitabine-based X-ray telescope (XRT) radiation (30 Gy); 75% of patients were resected, 95% had R0 resections and the median OS for those who completed all therapy was 34-months.^29^ Their subsequent study of cisplatin and gemcitabine followed by gemcitabine-based chemoradiation in 90 patients with remote procedure call (RPC) revealed an R0 resection rate of 96% and median OS of 31-months.^30^ Cloyd et al published a unique retrospective study utilizing propensity score weighted methodologies. The authors queried MD Anderson database to identify all patients who received pre-operative chemotherapy or CRT before pancreatectomy for anatomically resectable PDAC between 1999 and 2014. They concluded that the receipt of pre-operative CRT alone was associated with a higher rate of margin-negative resection (91% *vs.* 79%, *p*<0.01), lower rate of positive lymph nodes (53% *vs.* 23%, *p*<0.01), greater treatment effect, reduced incidence of locoregional recurrence (LR) (LR; 16% *vs.* 33%, *p*<0.01) but similar median overall survival (OS; 33.6 *vs.* 26.4-months, *p*=0.09) compared with systemic chemotherapy alone.^31^ Katz et al, report- ed wider special memorandum account (SMA) margin distance on histological examination on patients who receive pre-operative CRT.^32^ This suggests that the local effect of CRT may occur primarily through sterilization of the retroperitoneum.

### THE IMPACT OF SBRT ON THE TUMOR MICROENVIRONMENT

Both SBRT and SRS have been used effectively for the treatment of lung, liver, brain, prostate, and recurrent head and neck cancers, among others.^33–37^ Damage to tumor cell deoxyribonucleic acid (DNA) is thought to account for only part of the efficacy of hypofractionated regimens.^38^ Many studies indicate that in addition to the direct impact on DNA, the effects of high-dose radiation on the tumor microenvironment (TME) may play a role in tumor control by SBRT and stereotactic radiosurgery (SRS).^38–41^ Many studies indicate the effect of a single fraction or hypofractionated radiation therapy in the treatment of pancreatic tumor xenografts.

**Figure 1.**
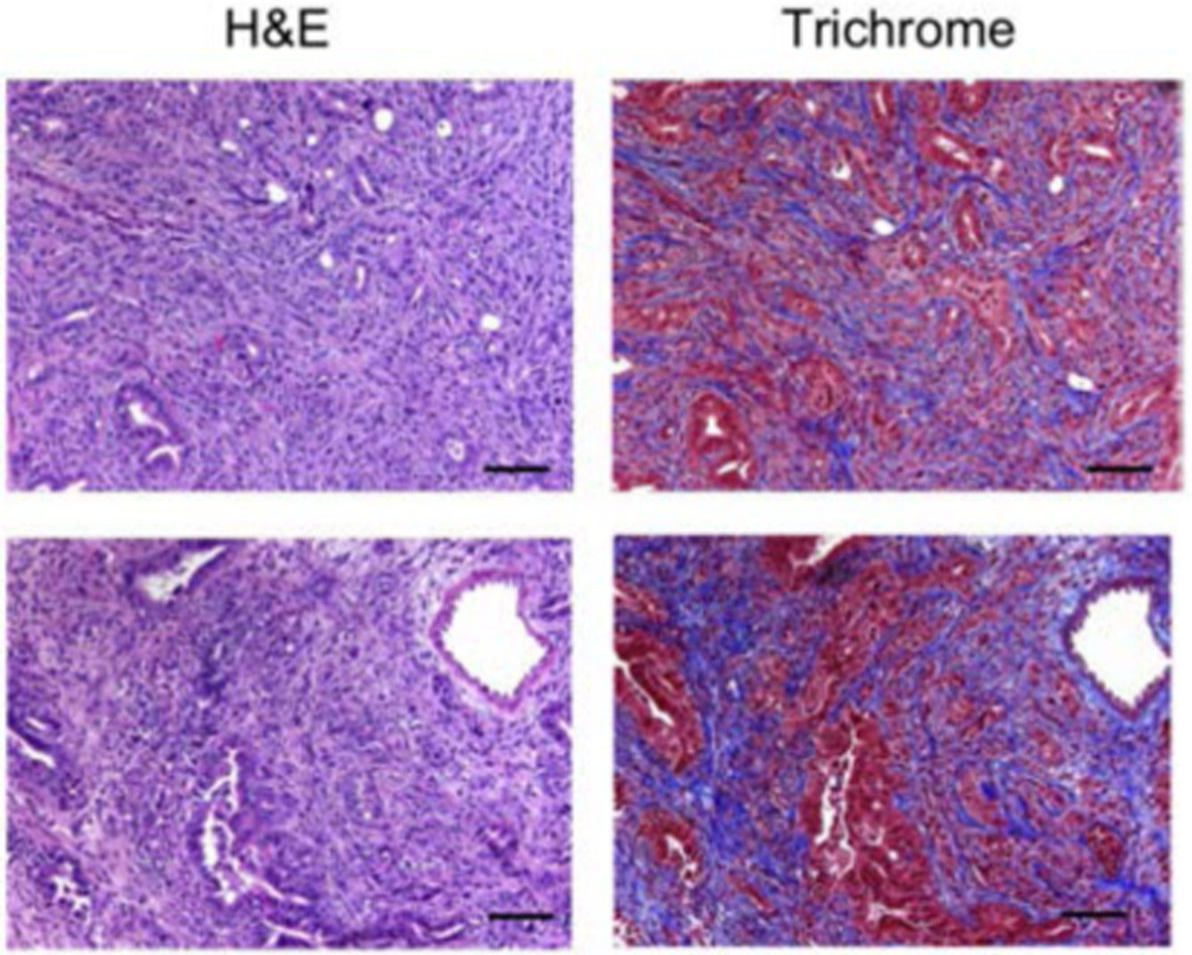
The Paradoxical Web of Pancreatic Cancer Tumor Microenvironment Hematoxylin and eosin (H&E) and trichrome staining of pancreatic tumors arising in two KPC mice recapitulating the dense collagen-rich stroma seen in human pancreatic adenocarcinoma tumors. Scale bars Z 100 mm.

In the stroma of human carcinomas, cancer-associated fibroblasts (CAFs) are the most abundant cell types and play a significant role in tumor cell growth, angiogenesis, and invasiveness (Figure 1).^42–46^ CAFs are also responsible for the deposition of key extracellular matrix (ECM) proteins (e.g., collagen, fibronectin, and laminin) as well as secreting ECM-degrading enzymes (e.g., matrix metalloproteinases),^42,43^ which promotes migration of CAFs and degradation of the ECM, allowing the invasion of tumor cells.^47^

*In vitro* studies have shown that fibroblasts develop an irreversible senescent phenotype when exposed to a dose>10 Gy of radiation, whereas low doses of radiation induce reversible DNA damage without growth arrest. Senescent fibroblasts release proteolytic enzymes, cytokines, growth factors, and reactive oxygen species, creating a protumorigenic environment.^48^ Radiation doses higher than 10 Gy per fraction are associated with severe vascular damage leading to the deterioration of the TME.^39,49^ Although endothelial cell damage has been shown to be a major factor in the biological mechanism of SBRT and SRS, this phenomenon is sometimes transient and may lead to neovasculogenesis *via* hypoxia-inducible factor (HIF)-1 induction.^49^ Baird et al reported pancreatic tumor regression through activation of type 1 interferon-dependent responses with a single dose of 10 Gy and co-treatment with *cGAMP* or *STING* (simulator of interferon genes) agonists that amplify the radiation-induced antitumor immune response.^50,51^ Type 1 interferons (interferon (IFN)-α and IFN-β) are important for activation of both innate and adaptive immune responses and are well-known for their role in viral immunity.^52^

Treatment of pancreatic tumor xenografts with radiation given as 4 Gy in 2 fractions resulted in a switchin tumor-infiltrating macrophages from a protumorigenic M2 phenotype to an antitumorigenic M1 phenotype.^53^ Likewise, increased infiltration of T-cells into tumors and tumor killing mediated by iNOS+M1 macro- phages through the expression of Type 1 T helper (TH1) cytokines have been reported in murine models of pancreatic cancer and melanoma after low-dose radiation treatment.^53,54^ Moreover, many studies have demonstrated M2 polarization after treatment with single high-dose and hypofractionated radiation regimens.^55–57^ Several clinical trials are underway to determine the effects of combination therapy with radiation and immune checkpoint inhibitors (Table 3).^58–60^

## CONCLUSION

SBRT has been shown to be safe and effective in pancreatic cancer patients. It offers several advantages over standard EBRT including increased patient convenience, reduced toxicities, and the ability to minimize delays in modern multi-agent chemotherapy. The ability of SBRT to convert patients with borderline and locally advanced tumors to resectable disease with higher percentage of negative resection margins may improve survival. Favorable SBRT outcomes for LAPC patients have paved the way for exploration of SBRT for resectable pancreatic cancer patients, with promising early results. The immunotherapeutic approach has very limited clinical activity to date in pancreatic cancer, it is still unclear how to optimally combine ablative radiation and immunotherapy, including optimal sequencing, radiation dose to effectively overcome the immunosuppressive pancreatic tumor microenvironment.

## Figures and Tables

**Table 1.** SBRT for Locally Advanced Pancreatic Cancer

Study	n	Dose Fractionation	Chemo	Local control	Survival	Toxicity
Koong et al^8^	6	25Gy in 1fx (73 Gy2)	None	100% @ 1 year	Median 8 mo	33% acute G3+
Chang et al^9^	77	25Gy in 1fx (73 Gy2)	Gemcitabine	84% @ 1 year	Median 12 mo	25% G2+ @ 1yr
Mahadevan et al^11^	39	24–36Gy in 5fx (30–50 Gy2)	Gemcitabine	85% crude	Median 20 mo	9% late G3+
Herman et al^13^	49	33Gy in 5fx (46 Gy2)	Gemcitabine	78% @ 1 year	Median 13.9 mo	12% acute G3+ 11% late G2+
Moningi et al^17^	88	25–33Gy in 5fx (31–46 Gy2)	Gemcitabine or FOLFIRINOX	61% @ 1 year	Median 18.4 mo	3% acute G3+ 6% late G2+

**Table 2.** SBRT for Borderline Resectable Pancreatic Cancer

Study	n	Dose Fractionation	Chemo	Survival	Conversion rate	R0	pCR	Toxicity
Chuong et al^12^	73 (78% BRPC)	25–50Gy in 5fx (31–83 Gy2)	GTX	Median 16.4m 72% @ 1 year	56%	97%	Not reported	0% acute G3+ 5% late G3+
Mellon et al^14^	159 (69% BRPC)	30–40 Gy in 5fx (40–60 Gy2	GTX	Median 19.2 m	51%	96%	7%	7% acute & late G3+
Rajagopalan et al ^18^	12 (58% BRPC)	36 Gy in 3fx (66 Gy2) 24 Gy in 1fx (68 Gy2)	Gemcitabine-Capecitabine	Median 47.2 m 92% @ 1 year	100%	92%	25%	0% acute G3+

**Table 3.** Ongoing Pancreatic Trials

Unresectable pancreatic cancer	NCT01926197
Borderline resectable pancreatic cancer	NCT01992705, NCT02308722, NCT01446458
Resectable pancreatic cancer	NCT03704662, NCT02347618, NCT02318095, NCT02208024, NCT01446458

## References

[1] Heestand GM, Murphy JD, Lowy AM. Approach to patients with pancreatic cancer without detectable metastases. J Clin Oncol.2015; 33: 1770–1778. doi: 10.1200/JCO.2014.59.7930 25918279

[2] Pollom EL, Alagappan M, von Eyben R, Single-versus multifraction stereotactic body radiation therapy for pancreatic adenocarcinoma: Outcomes and toxicity. Int J RadiatOncol Biol Phys. 2014; 90(4): 918–925. doi: 10.1016/j.ijrobp.2014.06.066 25585785

[3] Burris HA, Moore MJ, Andersen J, Improvements line therapy for patients with advanced pancreas cancer: A randomized trial. J Clin Oncol. 1997; 15: 2403–2413. doi: 10.1200/JCO.1997.15.6.2403 9196156

[4] Chauffert B, Mornex F, Bonnetain F, Phase III trial comparing intensive induction chemoradiotherapy (60 Gy, infusional 5-FU and intermittent cisplatin) followed by maintenance gemcitabine with gemcitabine alone for results of the 2000–01 FFCD/SFRO study. Ann Oncol. 2008; 19: 1592–1599. doi: 10.1093/annonc/mdn281 18467316

[5] Von Hoff DD, Ervin T, Arena FP, Increased survival in pancreatic cancer with nab-paclitaxel plus gemcitabine. N Engl J Med. 2013; 369: 1691–1703. doi: 10.1056/NEJMoa1304369 24131140 PMC4631139

[6] Conroy T, Desseigne F, Ychou M, FOLFIRINOX versus gemcitabine for metastatic pancreatic cancer. N Engl J Med. 2011; 364: 1817–1825. doi: 10.1056/NEJMoa1011923 21561347

[7] Mukherjee S, Hurt CN, Bridgewater J, Gemcitabinebased or capecitabine-based chemoradiotherapy for locally advanced pancreatic cancer (SCALOP): A multicentre, randomised, phase 2 trial. Lancet Oncol. 2013; 14: 317–326. doi: 10.1016/S1470-2045(13)70021–4 23474363 PMC3620899

[8] Koong AC, Le QT, Ho A, Phase I study of stereotactic radiosurgery in patients with locally advanced pancreatic cancer. Int J Radiat Oncol Biol Phys. 2004; 58: 1017–1021. doi: 10.1016/j.ijrobp.2003.11.004 15001240

[9] Chang DT, Schellenberg D, Shen J, Stereotactic radiotherapy for unresectable adenocarcinoma of the pancreas. Cancer. 2009; 115: 665–672. doi: 10.1002/cncr.24059 19117351

[10] Mahadevan A, Jain S, Goldstein M, Stereotactic body radiotherapy and gemcitabine for locally advanced pancreatic cancer. Int J Radiat Oncol Biol Phys. 2010; 78: 735–742. doi: 10.1016/j.ijrobp.2009.08.046 20171803

[11] Mahadevan A, Miksad R, Goldstein M, Induction gemcitabine and stereotactic body radiotherapy for locally advanced nonmetastatic pancreas cancer. Int J Radiat Oncol Biol Phys. 2011; 81: e615–622. doi: 10.1016/j.ijrobp.2011.04.045 21658854

[12] Chuong MD, Springett GM, Freilich JM, Stereotactic body radiation therapy for locally advanced and borderline resectable pancreatic cancer is effective and well tolerated. Int J Radiat Oncol Biol Phys. 2013; 86: 516–522. doi: 10.1016/j.ijrobp.2013.02.022 23562768

[13] Herman JM, Chang DT, Goodman KA, Phase 2 multi-institutional trial evaluating gemcitabine and stereotactic body radiotherapy for patients with locally advanced unresectable pancreatic adenocarcinoma. Cancer. 2015; 121: 1128–1137. doi: 10.1002/cncr.29161 25538019 PMC4368473

[14] Mellon EA, Hoffe SE, Springett GM, Long-term out- comes of induction chemotherapy and neoadjuvant stereotactic body radiotherapy for borderline resectable and locally advanced pancreatic adenocarcinoma. Acta Oncol. 2015; 54: 979–985. doi: 10.3109/0284186X.2015.1004367 25734581

[15] Chhabra A, Kaiser A, Regine WF, Chuong MD. The expanding role of stereotactic body radiation therapyfor pancreatic cancer: A review of the literature. Transl Cancer Res. 2015; 4(6): 659–670. doi: 10.3978/j.issn.2218-676X.2015.11.01

[16] Moningi S, Marciscano AE, Rosati LM, Stereotactic body radiation therapy in pancreatic cancer: The new frontier. Expert Rev Anticancer Ther. 2014; 14: 1461–1475. doi: 10.1586/14737140.2014.952286 25183386

[17] Moningi S, Dholakia AS, Raman SP, The role of stereotactic body radiation therapy for pancreatic cancer: A single-institution experience. Ann Surg Oncol. 2015; 22: 2352–2358. doi: 10.1245/s10434-014-4274-5 25564157 PMC4459890

[18] Rajagopalan MS, Heron DE, Wegner RE, Pathologic response with neoadjuvant chemotherapy and stereotactic body radiotherapy for borderline resectable and locallyadvanced pancreatic cancer. Radiat Oncol. 2013; 8: 254. doi: 10.1186/1748-717X-8-254 24175982 PMC4228466

[19] He J, Moningi S, Blair AB, Surgical outcomes of patients with pancreatic cancer treated with stereotactic body radiation therapy. J Clin Oncol. 2015; 33: abstr 341. doi: 10.1200/jco.2015.33.3_suppl.341

[20] Verbeke CS, Gladhaug IP. Resection margin involvement and tumour origin in pancreatic head cancer. Br J Surg. 2012; 99(8): 1036–1049. doi: 10.1002/bjs.8734 22517199

[21] Verbeke CS, Leitch D, Menon KV, McMahon MJ, Guillou PJ, Anthoney A. Redefining the R1 resection inpancreatic cancer. Br J Surg. 2006; 93: 1232–1237. doi: 10.1002/bjs.5397 16804874

[22] Menon KV, Gomez D, Smith AM, Anthoney A, Verbeke CS. Impact of margin status on survival following pancreatoduodenectomy for cancer: The Leeds Pathology Protocol (LEEPP). HPB (Oxford). 2009; 11: 18–24. doi: 10.1111/j.1477-2574.2008.00013.x 19590619 PMC2697870

[23] Esposito I, Kleeff J, Bergmann F, Most pancreatic cancer resections are R1 resections. Ann Surg Oncol. 2008; 15: 1651–1660. doi: 10.1245/s10434-008-9839-8 18351300

[24] Campbell F, Smith RA, Whelan P, Classification of R1 resections forpancreatic cancer: The prognostic relevance of tumour involvement within 1 mm of a resection margin. Histopathology. 2009; 55: 277–283. doi: 10.1111/j.1365-2559.2009.03376.x 19723142

[25] Jamieson NB, Foulis AK, Oien KA, Positive immobilization margins alone donot influence survival following pancreatico-duodenectomy for pancreatic ductal adenocarcinoma. Ann Surg. 2010; 251: 1003–1010. doi: 10.1097/SLA.0b013e3181d77369 20485150

[26] Verbeke CS, Gladhaug IP. Resection margin involvement and tumour origin in pancreatic head cancer. Br J Surg. 2012; 99: 1036–1049. doi: 10.1002/bjs.8734 22517199

[27] Colbert LE, Hall WA, Nickleach D, Switchenko J, Kooby DA, Landry JC. Chemoradiation therapy sequencing for resected pancreatic adenocarcinoma in the National Cancer Data Base. Cancer. 2014; 120(4): 499–506. doi: 10.1002/cncr.28530 24390739 PMC4380170

[28] Neoptolemos JP, Palmer DH, Ghaneh P, Comparison of adjuvant gemcitabine and capecitabine with gemcitabine monotherapy in patientswith resected pancreatic cancer (ESPAC-4): A multicentre, open-label, randomised, phase 3 trial. Lancet. 2017; 389(10073): 1011–1024. doi: 10.1016/S0140-6736(16)32409-6 28129987

[29] Evans DB, Varadhachary GR, Crane CH, Preoperative gemcitabine-based chemoradiation for patients with resectable adenocarcinoma of the pancreatic head. J Clin Oncol. 2008; 26: 3496–3502. doi: 10.1200/JCO.2007.15.8634 18640930

[30] Varadhachary GR, Wolff RA, Crane CH, Preoperative gemcitabine and cisplatin followed by gemcitabine-based chemoradiation for resectable adenocarcinoma of the pancreatic head. J Clin Oncol. 2008; 26: 3487–3495. doi: 10.1200/JCO.2007.15.8642 18640929

[31] Cloyd JM, Chen HC, Wang X, Chemotherapy versus chemoradiation as preoperative therapy for resectable pancreatic ductal adenocarcinoma: A propensity score adjusted analysis. Pancreas. 2019; 48(2): 216–222. doi: 10.1097/MPA.0000000000001231 30629022

[32] Katz MH, Wang H, Balachandran A, Effect of neoadjuvant chemoradiation and surgical technique on recurrence of localizedpancreatic cancer. J Gastrointest Surg. 2012; 16: 68–78; discussion 78–79. doi: 10.1007/s11605-011-1748-7 22065318

[33] Mahajan A, Ahmed S, McAleer MF, Post-operative stereotactic radiosurgery versus observation for completely resected brain metastases: A single-centre, randomised, controlled, phase 3 trial. Lancet Oncol. 2017; 18: 1040–1048. doi: 10.1016/S1470-2045(17)30414-X 28687375 PMC5560102

[34] Aridgides P, Bogart J. Stereotactic body radiation therapy for stage I non-small cell lung cancer. Thorac Surg Clin. 2016; 26: 261–269. doi: 10.1016/j.thorsurg.2016.04.008 27427521

[35] Qiu H, Moravan MJ, Milano MT, Usuki KY, Katz AW. SBRT for hepatocellular carcinoma: 8-year experience from a regional transplant center. J Gastrointest Cancer. 2018; 49(4): 463–469. doi: 10.1007/s12029-017-9990-1 28710606

[36] Kishan AU, King CR. Stereotactic body radiotherapy for low- and intermediate-risk prostate cancer. Semin Radiat Oncol. 2017; 27: 268–278. doi: 10.1016/j.semradonc.2017.02.006 28577834

[37] Baliga S, Kabarriti R, Ohri N, Stereotactic body radiotherapy for recurrent head and neck cancer: A critical review. Head Neck. 2017; 39: 595–601. doi: 10.1002/hed.24633 27997054

[38] Kim MS, Kim W, Park IH, Radiobiological mechanisms of stereotactic body radiation therapy and stereotactic radiation surgery. Radiat Oncol J. 2015; 33: 265–275. doi: 10.3857/roj.2015.33.4.265 26756026 PMC4707209

[39] Song CW, Lee YJ, Griffin RJ, Indirect tumor cell death after high-dose hypofractionated irradiation: Implications for stereotactic body radiation therapy and stereotactic radiation surgery. Int J Radiat Oncol Biol Phys. 2015; 93: 166–172. doi: 10.1016/j.ijrobp.2015.05.016 26279032 PMC4729457

[40] Park HJ, Griffin RJ, Hui S, Levitt SH, Song CW. Radiation-induced vascular damage in tumors: implications of vascular damage in ablative hypofractionated radiotherapy (SBRT and SRS). Radiat Res. 2012; 177: 311–327. doi: 10.1667/rr2773.1 22229487

[41] Brown JM, Carlson DJ, Brenner DJ. The tumor radiobiology of SRS and SBRT: Are more than the 5 R’s involved? Int J Radiat Oncol Biol Phys. 2014; 88: 254–262. doi: 10.1016/j.ijrobp.2013.07.022 24411596 PMC3893711

[42] Bhowmick NA, Neilson EG, Moses HL. Stromal fibroblasts in cancer initiation and progression. Nature. 2004; 432: 332–337. doi: 10.1038/nature03096 15549095 PMC3050735

[43] Kalluri R, Zeisberg M. Fibroblasts in cancer. Nat Rev Cancer. 2006; 6: 392–401. doi: 10.1038/nrc1877 16572188

[44] Orimo A, Weinberg RA. Stromal fibroblasts in cancer: a novel tumor-promoting cell type. Cell Cycle. 2006; 5: 1597–1601. doi: 10.4161/cc.5.15.3112 16880743

[45] Shimoda M, Mellody KT, Orimo A. Carcinoma-associated fibroblasts are a rate-limiting determinant for tumour progression. Semin Cell Dev Biol. 2010; 21: 19–25. doi: 10.1016/j.semc-db.2009.10.002 19857592 PMC2828545

[46] Mishra P, Banerjee D, Ben-Baruch A. Chemokines at the crossroads of tumor-fibroblast interactions that promote malignancy. J Leukoc Biol. 2011; 89: 31–39. doi: 10.1189/jlb.0310182 20628066

[47] Gaggioli C, Hooper S, Hidalgo-Carcedo C, Fibroblast- led collective invasion of carcinoma cells with differing roles for RhoGTPases in leading and following cells. Nat Cell Biol. 2007; 9: 1392–1400. doi: 10.1038/ncb1658 18037882

[48] Arnold KM, Flynn NJ, Raben A, The impact of radiation on the tumor microenvironment: Effect of dose and fractionation schedules. Cancer Growth Metastasis. 2018; 11: 1179064418761639. doi: 10.1177/1179064418761639 PMC5846913 29551910

[49] Maeda A, Chen Y, Bu J, Mujcic H, Wouters BG, DaCosta RS. In vivo imaging reveals significant tumor vascular dysfunction and increased tumor hypoxia-inducible factor-1α expression induced by high single-dose irradiation in a pancreatic tumor model. Int J Radiat Oncol Biol Phys. 2017; 97: 184–194. doi: 10.1016/j.ijrobp.2016.09.005 27816364

[50] Deng L, Liang H, Xu M, STING-dependent cytosolic DNA sensing promotes radiation-induced type I interferon-dependent antitumor immunity in immunogenic tumors. Immunity. 2014; 41: 843–852. doi: 10.1016/j.immuni.2014.10.019 25517616 PMC5155593

[51] Baird JR, Friedman D, Cottam B, Radiotherapy combined with novel STING-targeting oligonucleotides results in regression of established tumors. Cancer Res. 2016; 76: 50–61. doi: 10.1158/0008-5472.CAN-14-3619 26567136 PMC4703500

[52] Perry AK, Chen G, Zheng D, Tang H, Cheng G. The host type I interferon response to viral and bacterial infections. Cell Res. 2005; 15: 407–422. doi: 10.1038/sj.cr.7290309 15987599

[53] Prakash H, Klug F, Nadella V, Mazumdar V, Schmitz-Winnen-thal H, Umansky L. Low doses of gamma irradiation potentially modifies immunosuppressive tumor microenvironment by retuning tumor-associated macrophages: Lesson from insulinoma. Carcinogenesis. 2016; 37: 301–313. doi: 10.1093/carcin/bgw007 26785731

[54] Klug F, Prakash H, Huber PE, Low-dose irradiation pro- grams macrophage differentiation to an INOS(+)/M1 phenotype that orchestrates effective T cell immunotherapy. Cancer Cell. 2013; 24: 589–602. doi: 10.1016/j.ccr.2013.09.014 24209604

[55] Chiang CS, Fu SY, Wang SC, Irradiation promotes an m2 macrophage phenotype in tumor hypoxia. Front Oncol. 2012; 2: 89. doi: 10.3389/fonc.2012.00089 22888475 PMC3412458

[56] Okubo M, Kioi M, Nakashima H, M2-polarized macrophages contribute to neovasculogenesis, leading to relapse of oral cancer following radiation. Sci Rep. 2016; 6: 27548. doi: 10.1038/srep27548 PMC4897643 27271009

[57] Seifert L, Werba G, Tiwari S, Radiation therapy induces macrophages to suppress T-cell responses against pancreatic tumors in mice. Gastroenterology. 2016; 150: 1659.e5–1672.e5. doi: 10.1053/j.gastro.2016.02.070 PMC4909514 26946344

[58] Kang J, Demaria S, Formenti S. Current clinical trials testing the combination of immunotherapy with radiotherapy. J Immunother Cancer. 2016; 4: 51. doi: 10.1186/s40425-016-0156-7 27660705 PMC5028964

[59] Berberat PO, Kunzli BM, Gulbinas A, An audit of outcomes of a series of periampullary carcinomas. Eur J SurgOncol. 2009; 35: 187–191. doi: 10.1016/j.ejso.2008.01.030 18343082

[60] Lafaro KJ, Melstrom LG. The paradoxical web of pancreatic cancer tumor microenvironment. Am J Pathol. 2019; 189(1): 44–57. doi: 10.1016/j.ajpath.2018.09.009 30558722 PMC6315325

